# Dietary intake and growth of HIV exposed and unexposed 6–12 months old infants in South Africa

**DOI:** 10.1111/mcn.13740

**Published:** 2024-10-14

**Authors:** Phumudzo Tshiambara, Marinel Hoffman, Heather Legodi, Yusentha Balakrishna, Ute Feucht

**Affiliations:** ^1^ Department of Human Nutrition Faculty of Health Sciences University of Pretoria Pretoria South Africa; ^2^ Department of Consumer and Food Sciences Faculty of Natural and Agricultural Sciences University of Pretoria Pretoria South Africa; ^3^ Research Centre for Maternal, Fetal, Newborn and Child Health Care Strategies University of Pretoria Pretoria South Africa; ^4^ Research Unit for Maternal and Infant Health Care Strategies, South African Medical Research Council Cape Town South Africa; ^5^ Department of Human Nutrition and Dietetics Sefako Makgatho Health Sciences University Ga‐Rankuwa South Africa; ^6^ Biostatistics Research Unit, South African Medical Research Council Durban South Africa; ^7^ Department of Paediatrics Faculty of Health Sciences University of Pretoria Pretoria South Africa

**Keywords:** complementary feeding, dietary intake, growth, HIV exposure, infants, urban setting

## Abstract

Factors affecting the growth of HIV‐exposed‐uninfected (HEU) children are multi‐factorial, with limited information available on the dietary intake from 6 months. This study compared the dietary intake, micronutrient composition of breastmilk, and growth of HEU and HIV‐unexposed‐uninfected (HUU) infants aged 6 and 12 months in an urban setting. A repeated cross‐sectional study used structured questionnaires to collect socio‐demographic, dietary intake, food group data, and anthropometric measurements in the Siyakhula study. The HEU (48%) and HUU (52%) infants were included (total *n* = 181). At 6 months, HEU infants had lower weight‐for‐age z‐scores (WAZ) (−0.6 ± 1.1 vs. 0.1 ± 1.2; *p* < 0.001), length‐for‐age z‐scores (−0.8 ± 1.4 vs. −0.1 ± 1.2; *p* < 0.001), and mid‐upper‐arm circumference‐for‐age z‐scores (MUACAZ) (0.5 ± 1.1 vs. 1.0 ± 0.9; *p* < 0.001) than HUU infants. At 12 months, HEU infants had lower WAZ, MUACAZ, and weight‐for‐length z‐scores compared to HUU infants (*p* < 0.05). Stunting was found at 6 (15%) and 12 (12%) months in HEU infants. The micronutrient composition of breastmilk fed to both groups was similar. Breastfeeding rates were lower in HEU than in HUU infants at 6 (49% vs. 64%; *p* = 0.005) and 12 (24% vs. 46%; *p* = 0.002) months. Less than 3% of HEU and HUU infants achieved minimal dietary diversity scores at 12 months. Dietary intake of fat was similar in all breastfed infants, but iron and vitamin B12 were higher in non‐breastfed HEU infants at 12 months. HEU infants had lower breastfeeding rates than HUU infants. A lack of dietary diversity was found in all infants. Nutrition education and counselling in the complementary feeding phase are essential for optimal growth.

## Key messages

- HIV‐exposed‐uninfected (HEU) infants had lower growth than HIV‐unexposed‐uninfected (HUU) infants at 6 and 12 months, but HEU infants could catch up on growth at 12 months. Stunting and underweight were high in HEU infants.
- At 6 and 12 months, breastfeeding rates were lower in HEU than in HUU infants with a lack of continued breastfeeding
- Dietary diversity was low in HEU and HUU infants, but flesh food consumption was higher in HEU than in HUU infants
- Dietary intakes were higher in breastfed HEU than HUU infants; HEU infants had better dietary macro‐ and micronutrient intakes than HUU infants.

## Graphical abstract

HIV‐exposed‐uninfected (HEU) infants were stunted at 6 months but catch up on growth at 12 months. Dietary diversity was low in HEU and HIV‐unexposed‐uninfected (HUU) infants, but flesh consumption was higher in HEU than in HUU infants. With lower breastfeeding rates at 6 and 12 months.

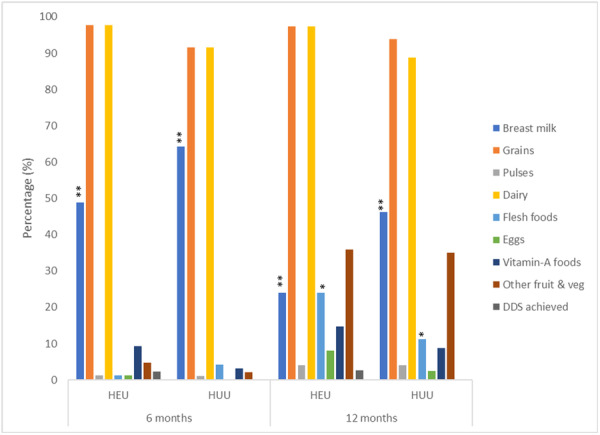

## 1 INTRODUCTION

Globally, undernutrition is a pressing public health concern, responsible for 45% of deaths in children under 5 years of age, mainly in low‐ and middle‐income countries (Prendergast & Evans, 2023). Undernutrition increases susceptibility to infections, compromising growth and development; but it can be prevented through appropriate and adequate dietary intake (World Health Organization [WHO], 2016).

In 2022, 39 million people worldwide were living with HIV, with 28.7 million receiving antiretroviral therapy (ART) (UNAIDS, 2022). In South Africa, 8.45 million (13.9%) people were living with HIV in 2022, with 24% being women of reproductive age (Stats, 2022). Mothers living with HIV (MLWH) fear vertical HIV transmission through breastfeeding, despite the risk reduction offered by ART (WHO, 2019a). Breastfeeding has many known benefits, even with maternal HIV infection (WHO, 2019b). South African breastfeeding rates are significantly lower than those in Sub‐Saharan Africa (45%) and globally (44%) (UNICEF, 2021b). The vertical transmission prevention of HIV programme and ART in 2002 and 2004, has reduced HIV‐related mortality and vertical transmission rates to 2.1%–2.6% in South Africa (Goga et al., 2016; Mmotsa et al., 2023; UNAIDS, 2011). With the ART coverage of 97% coverage among pregnant and breastfeeding women in 2023 (UNAIDS, 2024;). In SSA, more than one million children are exposed to HIV in utero, while remaining uninfected, referred to as HIV‐exposed‐uninfected (HEU) (Sugandhi et al., 2013).

Even in the context of HIV and ART, exclusive breastfeeding is recommended for the first 6 months, with the introduction of appropriate complementary foods and breastfeeding for 24 months or longer (WHO, 2019a). Complementary feeding means the introduction of a variety of appropriate, safe, and nutrient‐dense foods from 6 months of age while breastfeeding. It is crucial for optimal growth and development during the transition from exclusive breastfeeding (Dewey, 2013).

In Africa, inappropriate complementary feeding is commonly characterised by the early introduction of solid foods and a lack of dietary diversity (Faber, 2007; Kulwa et al., 2015). In Ethiopia (2017), only 26% of HEU infants were given appropriate complementary foods (Esubalew et al., 2018). South African studies reported it in 45%–88% of infants (Mugware et al., 2022; Sayed & Schönfeldt, 2020). Between 6 and 12 months, inappropriate complementary feeding can lead to increased nutritional needs, emphasising the importance of quality over quantity when breastfeeding is discontinued (Dewey, 2013). In South Africa, inappropriate complementary feeding is common due to lower dietary diversity with the predominant use of maize porridge (Faber et al., 2016).

Few studies have looked holistically at the influence of dietary intake, growth, and trace element composition of breast milk on the HEU or HUU infants between 6 and 12 months of age. The quality of complementary feeding in this age group is important, particularly in high HIV prevalence urban settings (Parker et al., 2013; Pedersen et al., 2016; Rahamon et al., 2013). Therefore, this study aims to compare the dietary intake, breast milk composition, and growth of HEU and HUU infants aged 6 and 12 months born in the study area.

## 2 METHODS AND MATERIALS

### 2.1 Study design and participants

This repeated cross‐sectional study is part of the Siyakhula study aimed at assessing factors impacting fetal and infant immunity and growth in HEU children, previously described elsewhere (Tshiambara et al., 2023). Mothers were screened for eligibility by trained research assistants in the participant's local language. Baseline mother–infant data were collected at birth from October 2018 at the Kalafong Provincial Tertiary Hospital, Gauteng Province. Dietary intake and anthropometric measurements were collected in 181 infants (HEU = 86 and HUU = 95) at 6 months and 155 infants (HEU = 75 and HUU = 80) at 12 months. Twenty‐six HEU and HUU infants were lost to follow‐up, because they relocated or could not complete measurements due to illnesses, and one infant became HIV‐infected by 12 months.

### 2.2 Data collection

#### 2.2.1 Socio‐demographic information

Socio‐demographic information, including maternal age, education level, employment status, alcohol use, and smoking, was collected after obtaining informed consent. Additionally, HIV information was collected, with all MLWH self‐reporting initiation of ART either before or during pregnancy.

#### 2.2.2 Anthropometry

Weight (calibrated digital scale; Seca 354), length (mechanical infantometer; Seca 416), head circumference, and mid‐upper‐arm‐circumference (non‐stretchable tape measure; KDS measure, model F10‐02DM 2 m) were measured while the infants were wearing minimal clothing. These measurements were provided at birth (except for mid‐upper‐arm‐circumference [MUAC]) as a baseline, as previously reported (Tshiambara et al., 2023), and, subsequently at 6 and 12 months. Weight‐for‐age z‐scores (WAZ), length‐for‐age z‐scores (LAZ), weight‐for‐length z‐scores (WLZ), head circumference‐for‐age z‐scores (HCAZ), and MUAC‐for‐age z‐scores (MUACAZ) were computed using the Intergrowth‐21st and WHO Anthro child growth standards v3.2.2 (Villar et al., 2014; WHO, 2006). Underweight (WAZ < 2 standard deviations [SD]), stunting (LAZ < 2 SD), wasting (WLZ < 2 SD), and overweight (WLZ > 2 SD) were determined for HEU and HUU infants, with reference to median values (WHO, 2006).

#### 2.2.3 Dietary intake

Mothers were privately interviewed at follow‐up visits by trained research assistants using their preferred local language. A 24‐h recall was used to collect detailed information on food consumption, time, type, amount, and preparation method as it is easy to administer. It has been previously used in dietary studies of infants aged 6 months (Faber et al., 2020; Walker et al., 2018). A standardised dietary kit containing samples of food and food containers, household utensils, and photographs, was used to measure the previous day's food intake. Mothers indicated the amount eaten by the infants using dry oats and a measuring cup to quantify the amount of food consumed by infants. Breast milk substitutes and commercial infant cereals were listed in dry and liquid amounts. In this study, the dietary intake of infants included complementary foods, other milk feeds, and breast milk substitutes, excluding breast milk, in line with other studies (Lane et al., 2019; Nyofane, 2020). This study also compared the dietary intakes of HEU and HUU infants who were breastfed and non‐breastfed, as the breast milk intake was not quantified.

#### 2.2.4 Breast milk composition

The macronutrient composition of human breast milk has been well described (Grote et al., 2016; Kim & Yi, 2020; Mabaya et al., 2022). This study explores micronutrient trace elements in human breast milk, which are crucial for infant growth; but are understudied due to differences in analytical methods between macronutrients and micronutrients. The study utilised Inductively Coupled Plasma Mass Spectroscopy (Hampel, Dror, et al., 2018) to analyse breast milk at 6 and 12 months post‐natally for trace elements, including iron, zinc, manganese, copper, and selenium. The MLWH and mothers not living with HIV (MnLWH) hand expressed (10 mL) in a labelled glass bottle with the participant number and study visit (6 or 12 months). Samples were stored in a sealed cooler box after hand expression and in a −80°C freezer until analysis. Breast milk samples were acidified with ultrapure nitric acid to 2% final concentrations and then dissolved. The ion detector converted the ions into electrical signals, and the results were interpreted using the MassHunter programme (Sun et al., 2015).

### 2.3 Data processing and analysis

Data were captured using Research Electronic Data Capture v 8.3.5 (Patridge & Bardyn, 2018). The South African Medical Research Council (SAMRC) Food Quantities Manual was used to convert reported food consumption quantities into weights for the 24‐h recall data. The SAMRC food finder software was used to quantify infants' macro‐ and micronutrient intake (SAFOODS, 2018). The study estimated nutrient densities (nutrient amount per 100 kcal) of a complementary diet and calculated a dietary diversity score using 24‐h recall data to determine the proportion of infants consuming a diet containing at least five of the eight food groups: breast milk, grains, legumes, dairy products, flesh foods, eggs, vitamin A‐rich fruits and vegetables, and other fruits and vegetables (DDS ≤ 4 vs. ≥5 groups) (WHO, 2021b). Descriptive statistics were used to present socio‐demographic information, dietary intake, anthropometric measurements, and HIV exposure. Continuous data was reported as means with SDs or medians with interquartile ranges (IQR), while categorical data was reported as frequencies and percentages. The Shapiro‐Wilk test was used to determine data normality, while the independent *t*‐test or Mann–Whitney *U* test and Pearson Chi‐squared/Fisher's exact tests were used to test differences in continuous variables and associations between categorical variables, respectively. All statistical analyses were carried out at a 5% level of significance using the Stata 16 programme.

### 2.4 Ethical considerations

The Siyakhula study was approved by the University of Pretoria's Faculty of Health Sciences Research Ethics Committee (Ref. no. 294/2017). All relevant information was shared with the mothers before data collection commenced. Mothers provided consent for themselves and their infants, and the Declaration of Helsinki criteria were followed. The Research Ethics Committees of the Faculty of Natural and Agricultural Sciences and the Faculty of Health Sciences at the same University approved this sub‐study (Ref. no. NAS063/2020). The study was conducted in accordance with the Declaration of Helsinki and approved by the Ethics Committee of the University of Pretoria (protocol code NAS063/2020 on 14 April 2024). Informed consent was obtained from all participants involved in the study.

## 3 RESULTS

### 3.1 Participant's characteristics

Maternal characteristics, stratified by HIV status, are presented in Table 1. Significant differences were found between MLWH and MnLWH in terms of age, education, gravidity, and parity, while gestational age did not differ (38.2 ± 1.5 vs. 38.3 ± 1.8 weeks; *p* = 0.293). More HEU infants were born with low birthweight than HUU infants (22% vs. 13%; *p* < 0.001), and the mean birth WAZ was lower in HEU than in HUU infants (−0.7 ± 0.9 vs. −0.2 ± 1.0; *p* = 0.003). Early introduction of solid foods was found in both HEU and HUU infants, with no significant difference in testing (*p* > 0.05). Water and Mabelle/maize meal soft porridge were introduced first by 84% and 76% of MLWH and MnLWH, respectively.

**Table 1:** Maternal characteristics stratified by maternal HIV status.

		MLWH	MnLWH	*p*‐Value
		*n* = 86	*n* = 95	
Age (years)^a^ mean ± SD		36.9 ± 8.6	31.3 ± 6.3	<0.001**
Age (years)^a^ *n* (%)	20–29	11 (12.8)	39 (41.1)	<0.001**
	30–39	55 (64.0)	45 (47.4)	
	≥40	20 (23.2)	11 (11.5)	
Education^a^ *n* (%)	Formal education, but without school completion^b^	55 (66.3)	31 (33.0)	<0.001**
	Completed secondary schooling	19 (22.9)	39 (41.5)	
	Tertiary education	9 (10.8)	24 (25.5)	
Employment (any)^a^ *n* (%)	Yes	41 (49.4)	43 (45.7)	0.738
Monthly income of the household (ZAR)^a^ *n* (%)	Do not know^c^	20 (23.3)	18 (18.9)	0.282
	R 0–4000	29 (33.8)	27 (28.4)	
	R 4001–8000	21 (24.4)	29 (30.5)	
	More than R 8001	16 (18.5)	21 (22.2)	
Child support grant^a^ *n* (%)	Yes	64 (74.4)	74 (77.9)	0.583
Marital status^a^ *n* (%)	Single/divorced/widow	60 (72.3)	74 (78.7)	0.412
	Married/cohabiting	23 (27.7)	20 (21.3)	
Access to water^a^ *n* (%)	Communal tap	21 (25.3)	19 (20.2)	0.534
	Inside yard	42 (50.6)	46 (48.9)	
	Inside house	20 (24.1)	29 (30.9)	
Access to electricity^a^ *n* (%)	Yes	79 (91.9)	90 (94.7)	0.437
Access to toilet^a^ *n* (%)	Flush toilet	54 (62.8)	64 (67.0)	0.519
	Pit latrine^d^	32 (37.2)	31 (33.0)	
Smoking^a^, ^e^ *n* (%)	Yes	3 (3.5)	3 (3.2)	n/a
Drinks alcohol (any) ^1,5^ *n* (%)	Yes	12 (14.0)	12 (12.6)	0.793
Mode of delivery^a^ *n* (%)	Vaginal delivery^f^	49 (57.0)	65 (68.4)	0.196
	Caesarean section	37 (43.0)	30 (31.6)	
Obstetric history median (IQR)	Gravidity	3 (2–4)	3 (2–3)	0.024*
	Parity	2 (1–3)	2 (1–2)	0.031*
	Previous pregnancy losses^g^	0 (0–1)	0 (0–1)	0.647

*Note*: Statistical analysis: To determine the difference in continuous data between mothers living and not living with HIV Mann–Whitney *U* test (non‐normally distributed) and for categorical data Pearson's Chi‐square test was used to determine the differences in mothers living with HIV and mothers not living with HIV.

Abbreviations: IQR, interquartile range; MLWH, mothers living with HIV; MnLWH, mothers not living with HIV; SD, standard deviation; ZAR, South African rand.

^a^ Excludes missing numbers.

^b^ Formal education = includes any primary and secondary schooling.

^c^ Do not know category excluded from analysis.

^d^ Pit toilet includes *n* = 2 MLWH with no access to a toilet in the yard.

^e^ At delivery and 6 months postpartum.

^f^ Includes assisted delivery.

^g^ Includes abortions, miscarriages, and terminations of pregnancy.

* *p* < 0.05

** *p* < 0.01.

### 3.2 Anthropometry measurements

Anthropometric measurements, z‐score indices, and nutritional classification of infants at 6 and 12 months of life by HIV‐exposure status are presented in Table 2. The birth WAZ of HEU infants was lower than HUU (−0.7 ± 0.9 vs. −0.2 ± 1.0; *p* = 0.003). At 6 months, HEU infants had significantly lower mean WAZ, LAZ, HCAZ, and MUACAZ than HUU infants. Among HEU infants, stunting (15%), underweight (9%), and wasting (4%) were found at age 6 months. At 12 months, HEU infants had lower mean WAZ, WLZ, and MUACAZ than HUU infants. Stunting rates were higher in HEU than in HUU infants (16% vs. 6%; *p* = 0.044) at 6 months.

**Table 2:** Anthropometric measurements, z‐score indices, and nutritional classifications of infants at 6 and 12 months of life by HIV exposure status.

	Age 6 months			Age 12 months		
	HEU infants	HUU infants	*p*‐Value	HEU infants	HUU infants	*p*‐Value
	(*n* = 86)	(*n* = 95)		(*n* = 75)	(*n* = 80)	
Age at visit (months)^a^ mean ± SD	6.1 ± 2.0	6.4 ± 1.8	0.383	12.3 ± 0.5	12.2 ± 0.5	0.160
Anthropometric measurements						
Weight^a^ (kg) mean ± SD	7.3 ± 0.9	7.8 ± 1.0	0.001**	9.1 ± 1.2	9.4 ± 1.3	0.106
Length (cm) mean ± SD	65.3 ± 3.5^a^	66.6 ± 2.8^a^	0.014*	74.4 ± 3.1	74.5 ± 2.7	0.704
Head circumference (cm) mean ± SD	43.5 ± 1.6^a^	43.9 ± 1.6^a^	0.106	46.5 ± 1.6	46.6 ± 1.6	0.655
Mid‐upper‐arm‐circumference^a^ (cm) mean ± SD	14.6 ± 1.3	15.2 ± 1.1	0.002**	15.6 ± 1.2	16.0 ± 1.3	0.075
Z‐score indices						
Weight‐for‐age z score^b^ mean ± SD	−0.6 ± 1.1	0.1 ± 1.2	<0.001**	−0.3 ± 1.1^a^	0.1 ± 1.2^a^	0.022*
Length‐for‐age z score^a^, ^b^ mean ± SD	−0.8 ± 1.4	−0.1 ± 1.2	<0.001**	−0.4 ± 1.3	−0.2 ± 1.1	0.308
Weight‐for‐length z score^a^, ^b^ mean ± SD	−0.1 ± 1.2	0.2 ± 1.1	0.074	−0.2 ± 1.2	0.2 ± 1.2	0.020*
Head circumference‐for‐age z‐score^a^, ^b^ mean ± SD	0.5 ± 1.2	0.9 ± 1.2	0.019*	0.6 ± 1.2	0.9 ± 1.1	0.069
Mid‐upper‐arm‐circumference‐for‐age z score^a^, ^b^ mean ± SD	0.5 ± 1.1	1.0 ± 0.9	<0.001**	0.8 ± 1.1	1.3 ± 1.1	0.025*
Nutritional classifications						
Underweight^c^ *n* (%)	8 (9.9)	3 (3.4)	0.082	4 (5.5)	4 (5.1)	>0.999
Stunted^d^ *n* (%)	13 (16.1)	5 (5.7)	0.044*	9 (12.3)	4 (5.1)	0.148
Wasted^e^ *n* (%)	3 (3.7)	2 (2.3)	0.670	4 (5.4)	3 (3.9)	0.714
Overweight^f^ *n* (%)	4 (4.9)	6 (6.8)	0.750	4 (5.5)	6 (7.8)	0.747

*Note*: Statistical analysis: Independent *t*‐test was used for continuous normally distributed data and Mann–Whitney *U* test was used for continuous that was not normally distributed; Pearson's Chi‐square/Fisher's exact test was used for categorical data to determine the differences in HEU and HUU infants.

Abbreviations: HEU, HIV‐exposed‐uninfected; HUU, HIV‐unexposed‐uninfected; SD, standard deviation.

^a^ Non‐normally distributed data.

^b^ Sex‐normalised z‐scores indices at ages 6 and 12 months were computed using WHO Anthro software of 2010.

^c^ Underweight from weight‐for‐age z‐scores <−2.

^d^ Stunted from length‐for‐age z‐scores <−2.

^e^ Wasted from weight‐for‐length z‐scores (WLZ) <−2.

^f^ Overweight from WLZ >+2.

* *p* < 0.05

** *p* < 0.01.

### 3.3 Food groups

**Figure 1:**
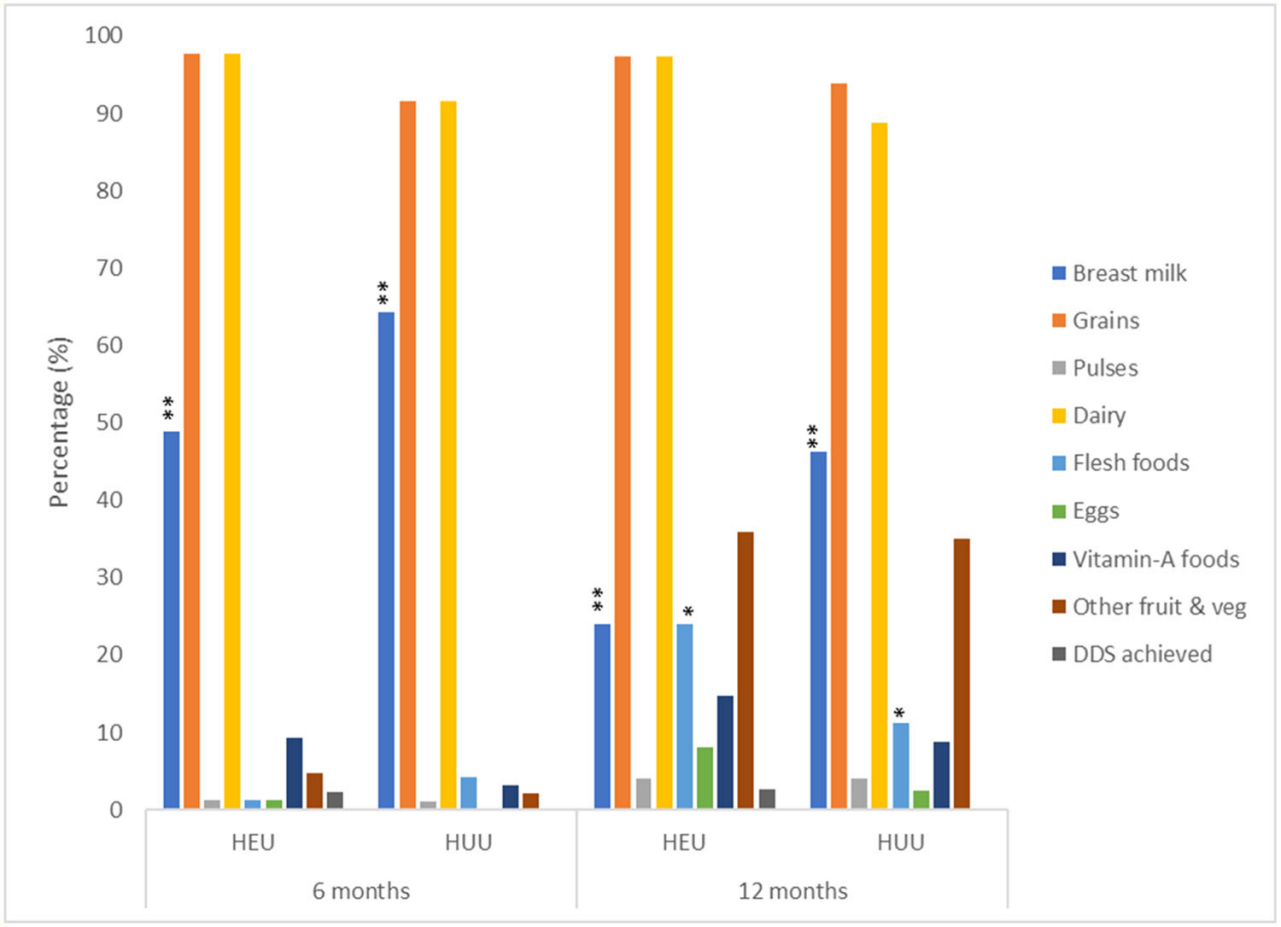
Food group consumption (any intake) and dietary diversity score of HEU and HUU infants at 6 and 12 months. HEU, HIV‐exposed‐uninfected; HUU, HIV‐unexposed‐uninfected. Statistical analysis: Pearson's Chi‐square/Fisher's exact test was used to analyse categorical data; a *p*‐value of *p < 0.05; **p < 0.01 shows significant differences in HEU and HUU groups.

Food groups consumed by HEU and HUU infants at 6 and 12 months are presented in Figure 1. A smaller proportion of HEU consumed any breast milk at 6 (*p* = 0.005) and 12 months (*p* = 0.002) than HUU infants. At 12 months, flesh foods consumption (any consumption thereof) was low overall, but higher among HEU than HUU infants (24% vs. 11%; *p* = 0.046). Dietary diversity was low in this study, with ≤3% having achieved minimal DDS at 12 months.

### 3.4 Breast milk composition

Trace elements composition of breast milk fed to HEU and HUU infants at 6 and 12 months is presented in Table 3. No significant differences were found in breast milk composition in MLWH and MnLWH (*p* > 0.05) at 6 and 12 months.

**Table 3:** Trace elements (mg/L) in human breast milk nutrient composition in MLWH and MnLWH at 6 and 12 months.

Trace elements (Median [IQR])	At 6 months post‐partum			At 12 months post‐partum		
	MLWH	MnLWH		MLWH	MnLWH	
	*n* = 16	*n* = 32	*p*‐Value	*n* = 5	*n* = 16	*p*‐Value
Iron	1.1 (0.5–1.5)	1.5 (0.9–2.3)	0.066	1.7 (1.0–2.1)	1.9 (1.6–2.3)	0.386
Zinc	6.6 (4.6–8.4)	5.3 (3.4–7.3)	0.182	6.4 (5.8–6.9)	4.2 (3.0–6.0)	0.052
Manganese	0.0 (0.0–0.0)	0.0 (0.0–0.0)	0.983	0.0 (0.0–0.0)	0.0 (0.0–0.1)	0.836
Copper	0.9 (0.8–1.2)	1.2 (0.8–1.5)	0.444	0.9 (0.7–1.1)	1.0 (0.8–1.2)	0.710
Selenium	0.1 (0.1–0.1)	0.1 (0.1–0.1)	0.197	0.1 (0.1–0.1)	0.1 (0.1–0.1)	0.836

*Note*: Statistical analysis: Mann–Whitney *U* test was used to determine the differences in continuous data.

Abbreviations: IQR, interquartile range; MLWH, mothers living with HIV; MnLWH, mothers not living with HIV.

### 3.5 Dietary intake of the infants

The dietary intake of breastfed and non‐breastfed HEU and HUU infants is presented in Table 4. Dietary intakes of protein (*p* = 0.014) and vitamin B12 (*p* = 0.010) were higher in breastfed HEU than HUU infants at 12 months. Non‐breastfed HEU infants had higher nutrient intake than HUU infants at 12 months (*p* < 0.05).

**Table 4:** Total intake of breastfed and non‐breastfed HEU and HUU infants at 6 and 12 months.^a^

	6 months						12 months					
	Partially breastfed infants			Non‐partially breastfed infants			Breastfed infants			Non‐breastfed infants		
	HEU (*n* = 42)	HUU *n* = 65	*p*‐Value	HEU *n* = 42	HUU *n* = 27	*p*‐Value	HEU *n* = 18	HUU *n* = 37	*p*‐Value	HEU *n* = 54	HUU *n* = 38	*p*‐Value
Protein^b^ (9.1 & 11 g)	9.0 (4.4–16.2)	7.5 (4.6–12.8)	0.522	14.8 (11.2–23.1)	18.8 (14.6–27.6)	0.079	16.6 (8.7–28.9)	11.7 (6.0–14.9)	0.014*	22.0 (15.8–29.6)	15.3 (10.2–23.5)	0.008**
Fat^b^ (30 g)	5.7 (2.3–12.2)	4.5 (1.8–14.8)	0.964	17.4 (12.7–25.5)	23.4 (18.2–35.2)	0.059	11.9 (8.2–26.0)	7.4 (4.3–14.0)	0.140	19.0 (13.1–26.7)	15.8 (8.3–23.4)	0.047*
Carbohydrates^b^ (95 g)	68 (35–148)	65 (32–102)	0.604	96 (54–120)	126 (97–182)	0.028*	92 (66–140)	85 (58–107)	0.249	125 (86–169)	95 (62–138)	0.013*
Calcium^b^ (260 mg)	166 (52–436)	204 (78–417)	0.566	479 (333–667)	653 (467–1119)	0.039*	194 (72–325)	131 (61–240)	0.258	433 (278–696)	312 (115–455)	0.014*
Iron^c^ (11 mg)	6.2 (2.2–14.9)	7.4 (3.2–12.0)	0.931	8.8 (5.8–13.0)	9.8 (6.8–18.6)	0.212	4.4 (2.4–7.7)	4.1 (3.1–5.4)	0.701	8.9 (5.6–11.6)	5.9 (3.4–8.2)	0.026*
Zinc^c^ (3 mg)	3.1 (1.3–5.8)	3.4 (1.5–6.9)	0.951	6.4 (4.1–8.5)	6.3 (5.3–9.7)	0.305	3.3 (1.9–7.6)	2.6 (1.9–4.3)	0.337	5.6 (4.1–7.1)	4.0 (2.1–5.9)	0.038*
Vitamin A^b^ (500 mcg)	362 (152–1121)	486 (272–800)	0.847	609 (431–999)	1063 (592–1437)	0.077	259 (157–433)	236 (158–359)	0.332	516 (276–793)	404 (171–631)	0.087
Vitamin B12^b^ (0.5 mcg)	0.2 (0.0–0.5)	0.4 (0.1–1.0)	0.056	1.0 (0.7–1.6)	1.2 (0.7–1.7)	0.375	0.6 (0.3–1.2)	0.2 (0.1–0.4)	0.010**	1.0 (0.5–1.6)	0.6 (0.3–0.9)	0.004**
Pantothenic acid^b^ (1.8 mg)	0.5 (0.2–1.4)	1.2 (0.2–2.5)	0.098	3.7 (2.4–5.0)	3.9 (2.7–5.4)	0.487	1.3 (0.6–2.1)	1.2 (0.6–2.2)	0.864	3.4 (1.7–5.4)	2.1 (0.7–4.5)	0.023*
Vitamin C^b^ (50 mg)	61 (20–138)	68 (36–111)	0.927	84 (52–127)	135 (98–181)	0.029*	45 (26–87)	25 (15–44)	0.051	67 (33–110)	49 (17–77)	0.087

*Note*: Dietary reference intakes. Statistical analysis: Mann–Whitney *U* test was used to determine the differences in continuous data.

Abbreviations: g, gram; HEU, HIV‐exposed‐uninfected (born to mothers living with HIV); HUU, HIV‐unexposed‐uninfected (born to mothers not living with HIV); IQR, interquartile range; mg, milligram; mcg, microgram; RE, retinol equivalence.

^a^ Total intake includes formula milk feeds, other milk feeds, and complementary foods (but excludes breast milk) and is presented as median (IQR).

^b^ Dietary reference intakes at 6–12 months.

^c^ Estimated average requirements at 6–12 months.

* *p* < 0.05

** *p* < 0.01.

## 4 DISCUSSION

We compared dietary intake, breast milk composition, and growth in HEU and HUU infants at 6 and 12 months of age. The MLWH had lower school completion rates than MnLWH, and education is a cost‐effective strategy for HIV prevention, as educated mothers are more likely to receive correct information, thereby reducing the risk of infection (Hargreaves et al., 2008).

HEU infants had higher low birthweight and lower WAZ and LAZ at birth and 6 months compared to HUU infants, a trend consistent with other African countries (Chalashika et al., 2017; Le Roux et al., 2019; Pillay et al., 2021). Stunting (HEU: 15% vs. HUU: 5%) and underweight (HEU: 9% vs. HUU: 3%) were noted in HEU and HUU infants at age 6 months in agreement with studies conducted in Botswana and Uganda (Chalashika et al., 2017; Osterbauer et al., 2012). Although HEU infants had lower LAZ than HUU infants at birth, HEU infants were able to catch up on growth, supporting the literature related to catch‐up growth (Le Roux et al., 2019; Slogrove et al., 2017). Lower WLZ was found in HEU compared to HUU infants at 12 months in our study, with similar findings reported in Zimbabwe (Omoni et al., 2017). Growth monitoring and promotion, including nutrition education and complementary feeding counselling, are crucial for improving HEU infants' growth and preventing stunting (Ara et al., 2019; Lassi et al., 2013; Prendergast et al., 2019).

Concerningly, we found early cessation of breastfeeding and introduction of complementary foods in both MLWH and MnLWH. These results corroborate with other African studies (Faber et al., 2020; Mugware et al., 2022; Tshiambara et al., 2023). Breastfeeding rates were lower in HEU than in HUU infants at 6 (49% vs. 64%; *p* = 0.005) and 12 (24% vs. 46%; *p* = 0.002) months, with similar findings reported in Rwandan HEU infants (Lane et al., 2019). Despite the recommendations and benefits of breastfeeding, stigma, culture, gravidity, and parity, past supply of BMS in the prevention of vertical transmission of HIV initiatives and lack of knowledge still influence the breastfeeding practices of MLWH, resulting in MLWH less likely to breastfeed (Neves et al., 2020; Rossouw et al., 2016; Tshiambara et al., 2023; Wanjohi et al., 2016). Breastfeeding is crucial for optimal growth and development, and raising awareness about its importance through nutrition education and counselling is essential for mothers, caregivers, and family members throughout the first 1000 days of life.

Although the practice of early introduction to solid foods is common in Africa, it is concerning as it may lead to undernutrition (WHO, 2021b). This study highlights the need for increased education and attention on age‐appropriate, diverse complementary foods for infants, as the DDS rate was low at 12 months, similar to other African studies (Mugware et al., 2022; Yisak et al., 2020). These results are concerning as they indicate possible inadequate micronutrient intake in most infants. Although flesh food consumption was low in both HEU and HUU infants, it was considerably lower in HUU infants (24% vs. 11%; *p* = 0.046) at 12 months in line with another South African study (17% infants) (Faber, 2007). Flesh foods are rich in iron but expensive. Raising awareness of healthy alternatives is beneficial in high HIV and anaemia prevalence. (Stats, 2022; WHO, 2021a).

The human breast milk composition of macronutrients has been previously described (Kim & Yi, 2020), but limited information exists on the composition of trace elements in human breast milk, which plays a crucial role in supporting macronutrient absorption and optimal growth and development of infants (Mahan & Raymond, 2016; Pedersen et al., 2016). In this study, iron and zinc human breast milk compositions in MLWH and MnLWH were similar at 6 and 12 months, contradicting previous studies with larger sample sizes and geographical areas (Bzikowska‐Jura et al., 2021; Hampel, Shahab‐Ferdows, et al., 2018; Nakamori et al., 2009). The differences may be attributed to factors such as sample size, geographical area, time of breast milk expression, and analytical methods used (Rios‐Leyvraz & Yao, 2023). Dewey (2001) indicated that iron and zinc composition are low during infancy, especially from 4 months onward; although no significant differences were found, MLWH and MnLWH had higher amounts of these nutrients. This may be due to factors affecting breast milk composition like quantification, lactation stage, body composition, and maternal dietary intake. Although breast milk intake quantification accuracy has concerns and requires complex analysis (Berube et al., 2018), other studies estimated an intake of 675 ml for partially breastfed infants at age 6–11 months (Faber et al., 2020; Swanepoel et al., 2019). This has policy implications and requires more studies with larger sample sizes in urban settings. The MLWH should be encouraged to breastfeed their infants while adhering to ART, as they have adequate breast milk to feed HEU infants without compromising their muscle mass (Mulol & Coutsoudis, 2016).

Inadequate dietary intake is an immediate determinant of undernutrition (UNICEF, 2021a). Dietary fat intake was low in breastfed and non‐breastfed HEU and HUU infants at 12 months. Low fat intakes were reported in Malawian HEU and South African infants (Faber et al., 2016; Parker et al., 2013). The findings are concerning as dietary fat is crucial for growth, development, energy provision, fat‐soluble vitamin absorption, and brain development (Mahan & Raymond, 2016). Non‐breasted HEU had higher dietary carbohydrate intakes than HUU infants at 12 months (*p* = 0.013). Higher intakes of carbohydrates were reported in African studies (Musakwa et al., 2020; Parker et al., 2013; Swanepoel et al., 2019) with sample size, age group, and HIV exposure status yielding different results from our study. Dietary protein intake is important for muscle strengthening and supporting the immune system (Mahan & Raymond, 2016). Breastfed and non‐breastfed HEU had higher protein intake than HUU infants at 12 months, with similar findings reported in Rwandan (Lane et al., 2019) and South African infants (Faber et al., 2016). Intakes of carbohydrate and protein intakes have been positively associated with weight and length gain in infants (Olga et al., 2023), suggesting a need to encourage mothers to breastfeed. Nutrition education and demonstrations can be effective interventions for all infants, including HUU, to encourage breastfeeding and appropriate complementary foods. Education is needed on affordable and easily accessible protein‐rich sources, such as lentils, eggs, and peanut butter.

Breastfed HEU and HUU infants had similar but low intakes of vitamin A at 12 months (500 μg/day), in line with Malawian breastfed HEU children and South African infants (Faber et al., 2016; Parker et al., 2013). South Africa has implemented a supplementation programme to prevent Vitamin A deficiency in children aged 6–59 months, as it is prevalent in children under 5 years old (Saitowitz et al., 2001). Education on affordable vitamin A‐rich foods and encouragement of mothers and caregivers to initiate home vegetable gardens to increase access to and consumption of these foods will be essential.

At 12 months, vitamin B12 dietary intake (0.5 μg/day) in breastfed HEU infants was higher than in HUU infants. This is concerning but expected as vitamin B12 is found in flesh foods and is essential for brain and nervous system nourishment as well as deoxyribonucleic acid formation (Mahan & Raymond, 2016). Eggs are an affordable and alternative source of vitamin B12 to flesh foods, but the consumption was very low (HEU = 8% vs. HUU = 2.5%) in our study. Faber et al. (2022) in South Africa found improved vitamin B12 intake after 3 months of daily egg consumption from 6 to 9 months, suggesting nutrition education and counselling could be beneficial.

Calcium is crucial for bone development and immune system support, and a lack of intake for 6 months can lead to rickets and stunting (Mahan & Raymond, 2016). Despite no significant differences between breastfed HEU and HUU infants, dietary calcium intake was low (<260 mg/day) at 12 months. Similar findings were reported in Rwandan infants (Lane et al., 2019) and Malawian HEU children. (Parker et al., 2013), Breast milk is recommended for optimal growth and development, but mothers should be advised on the proper handling and administration of a time‐consuming and expensive substitute (WHO, 2016). Education on local and affordable calcium‐rich sources will be beneficial to supplement breast milk.

No significant differences were found in the dietary iron intake, yet low in breastfed HEU and HUU infants at 12 months (<11 mg/day). Dietary intake of iron is important for optimal growth and development and the low intake may lead to anaemia. Nutrition education and counselling should be provided to MnLWH infants to include affordable iron‐rich foods, such as flesh foods, eggs, and lentils, in their diet to reduce the risk of anaemia at 12 months. Tshiambara et al. (2023), in the same study population, reported high consumption of miscellaneous food products that may inhibit iron absorption, using a 7‐day food frequency questionnaire. Discouraging the consumption of these products and encouraging families to start home vegetable gardens will be beneficial in this setting. Limited literature exists on dietary intakes and growth of HEU and HUU infants during complementary feeding phases, with HEU data often lacking comparison groups and older children. (Kulwa et al., 2015; Musakwa et al., 2020; Williams et al., 2016).

The strength of our study lies in detailed data on infant complementary feeding practices (food groups, dietary diversity, and dietary intake) and repeat anthropometric measurements collected by trained field workers to ensure quality control and validity. Our study also determined the composition of breast milk with a special focus on trace elements, a control group (HUU infants), and a similar sample size in the HEU and HUU groups. The limitations of our study include relatively small sample size, use of a single 24‐h recall, excluding breast milk intakes in the dietary intakes due to lack of quantification, and inability to make comparisons with the reference intakes due to the type of study. Future studies should include larger sample sizes, additional visits, multiple recalls, food diaries, and further regression models based on infant sex to better understand HEU infants' long‐term growth trajectories.

## 5 CONCLUSION

The study compared the dietary intakes, and growth of African HEU and HUU infants during the complementary feeding phase. HEU infants had lower z‐scores and breastfeeding rates but had higher intakes of flesh products. Non‐breastfed HUU infants had lower fat and iron intake compared to non‐breastfed HEU infants. These findings can guide nutrition policies and programmes to ensure optimal growth and development of all infants, especially during the complementary feeding period, regardless of HIV status.

## AUTHOR CONTRIBUTIONS

Phumudzo Tshiambara, Marinel Hoffman, Heather Legodi, and Ute Feucht involved in conceptualising the study. Phumudzo Tshiambara, Marinel Hoffman, and Ute Feucht designed the methodology. Formal analysis of the article was conducted by Yusentha Balakrishna and Phumudzo Tshiambara. Data curation and writing—original draft preparation were done by Phumudzo Tshiambara. Review and editing was conducted by Phumudzo Tshiambara, Heather Legodi, Marinel Hoffman, Yusentha Balakrishna and Ute Feucht. Visualisation of the data was taken care by Phumudzo Tshiambara, figure by Yusentha, and project administration was handled by Phumudzo Tshiambara.

## CONFLICT OF INTEREST STATEMENT

The authors declare no conflict of interest.

## Data Availability

Data that support the findings of this study are available on request from the corresponding author. The data are not publicly available due to privacy or ethical restrictions. The data supporting the findings will be made accessible upon request from the primary investigator of the Siyakhula study (Ute Feucht) following an embargo period to allow for the conclusion of the research and publication of findings.
